# The pan-BCL-2-blocker obatoclax (GX15-070) and the PI3-kinase/mTOR-inhibitor BEZ235 produce cooperative growth-inhibitory effects in ALL cells

**DOI:** 10.18632/oncotarget.18810

**Published:** 2017-06-28

**Authors:** Gabriele Stefanzl, Daniela Berger, Sabine Cerny-Reiterer, Katharina Blatt, Gregor Eisenwort, Wolfgang R. Sperr, Gregor Hoermann, Karin Lind, Alexander W. Hauswirth, Peter Bettelheim, Heinz Sill, Junia V. Melo, Ulrich Jäger, Peter Valent

**Affiliations:** ^1^ Department of Internal Medicine I, Division of Hematology & Hemostaseology, Medical University of Vienna, Vienna, Austria; ^2^ The Ludwig Boltzmann Cluster Oncology, Medical University of Vienna, Vienna, Austria; ^3^ Department of Laboratory Medicine, Medical University of Vienna, Vienna, Austria; ^4^ Department of Internal Medicine, Division of Hematology, Medical University of Graz, Graz, Austria; ^5^ Division of Laboratory Medicine, Elisabethinen Hospital Linz, Linz, Austria; ^6^ Faculty of Health and Medical Sciences, University of Adelaide, Adelaide, Australia

**Keywords:** ALL, targeting-concepts, BCL-2 family members, PI3-Kinase, mTOR

## Abstract

Acute lymphoblastic leukemia (ALL) is characterized by leukemic expansion of lymphoid blasts in hematopoietic tissues. Despite improved therapy only a subset of patients can be cured. Therefore, current research is focusing on new drug-targets. Members of the BCL-2 family and components of the PI3-kinase/mTOR pathway are critically involved in the regulation of growth and survival of ALL cells. We examined the effects of the pan-BCL-2 blocker obatoclax and the PI3-kinase/mTOR-inhibitor BEZ235 on growth and survival of ALL cells. In ^3^H-thymidine uptake experiments, both drugs suppressed the *in vitro* proliferation of leukemic cells in all patients with Philadelphia chromosome-positive (Ph^+^) ALL and Ph^−^ ALL (obatoclax IC_50_: 0.01-5 μM; BEZ235, IC_50_: 0.01-1 μM). Both drugs were also found to produce growth-inhibitory effects in all Ph^+^ and all Ph^−^ cell lines tested. Moreover, obatoclax and BEZ235 induced apoptosis in ALL cells. In drug-combination experiments, obatoclax and BEZ235 exerted synergistic growth-inhibitory effects on ALL cells. Finally, we confirmed that ALL cells, including CD34^+^/CD38^−^ stem cells and all cell lines express transcripts for PI3-kinase, mTOR, BCL-2, MCL-1, and BCL-xL. Taken together, this data shows that combined targeting of the PI3-kinase/mTOR-pathway and BCL-2 family-members is a potent approach to counteract growth and survival of ALL cells.

## INTRODUCTION

Acute lymphoblastic leukemia (ALL) is a life-threatening hematopoietic malignancy defined by abnormal proliferation and accumulation of lymphoid blast cells in lympho-hematopoietic organs including the bone marrow (BM) [1–4]. The course and prognosis in patients with ALL depend on disease-specific factors, including molecular abnormalities, and patient-specific variables such as age, co-morbidities, and response to initial therapy [1–6]. In a substantial subset of patients (roughly 20-30%) leukemic cells exhibit the Philadelphia-chromosome (Ph) and the related fusion gene, *BCR-ABL1* [1–6]. Before BCR-ABL1 tyrosine kinase inhibitors (TKI) were introduced in the treatment of Ph^+^ ALL, these patients had a poor overall outcome compared to those with Ph^−^ ALL [5, 6]. More recently, however, treatment-responses and the prognosis of patients with Ph^+^ ALL improved tremendously, which can be explained by the beneficial effects of novel drugs, especially BCR-ABL1 TKI such as imatinib [7–12]. In fact, imatinib is efficacious in the majority of patients with newly diagnosed Ph^+^ ALL, and can elicit meaningful effects even in patients with drug-resistant or relapsed ALL, especially when applied in combination with chemotherapy or allogeneic stem cell transplantation (SCT) [7–13]. Moreover, second- and third generation BCR-ABL1 blockers, such as dasatinib, nilotinib, or ponatinib, are available and may induce clinical responses in Ph^+^ ALL even when additional drug-resistant mutants are found [14–17]. Ponatinib exerts anti-leukemic effects even when ALL cells display the T315I mutant of BCR-ABL1 [17]. Nevertheless, not all ALL patients respond to treatment with conventional anti-leukemic drugs or BCR-ABL1 TKI. Therefore, SCT is often recommended for drug-resistant patients and those who have high risk ALL [18–21]. However, despite SCT and the use of novel TKI, not all ALL patients can be cured, and furthermore not all patients are eligible for SCT. Therefore, current research is focusing on the development of new concepts and novel agents or drug-combinations that can overcome resistance.

Several different pro-oncogenic pathways and survival-related molecules play an important role in the viability and proliferation of neoplastic cells in patients with ALL. The phosphatidylinositide 3 (PI3)-kinase/mechanistic target of rapamycin (mTOR) pathway has recently been described as a critical driver of oncogenesis in ALL [22–25]. Anti-apoptotic molecules contributing to survival of ALL cells include the heat shock proteins, epigenetic targets, and certain members of the BCL-2 family [26–30]. More recent data suggest that inhibitors of PI3-kinase, mTOR, and BCL-2 family members can counteract growth of ALL cells *in vitro* [26, 27, 30–32]. In addition, first clinical studies performed with PI3-kinase blockers and the BCL-2 family blocker venetoclax have shown promising results in lymphoid leukemias [33–35]. In the current study, we examined the effects of two drugs, one directed against the PI3-kinase/mTOR pathway (BEZ235) and the other directed against several different anti-apoptotic members of the BCL-2 family (obatoclax), on growth and survival of ALL cells.

## RESULTS

### ALL cells express BCL-2 family members, PI3-kinase, and mTOR

**Figure 1:**
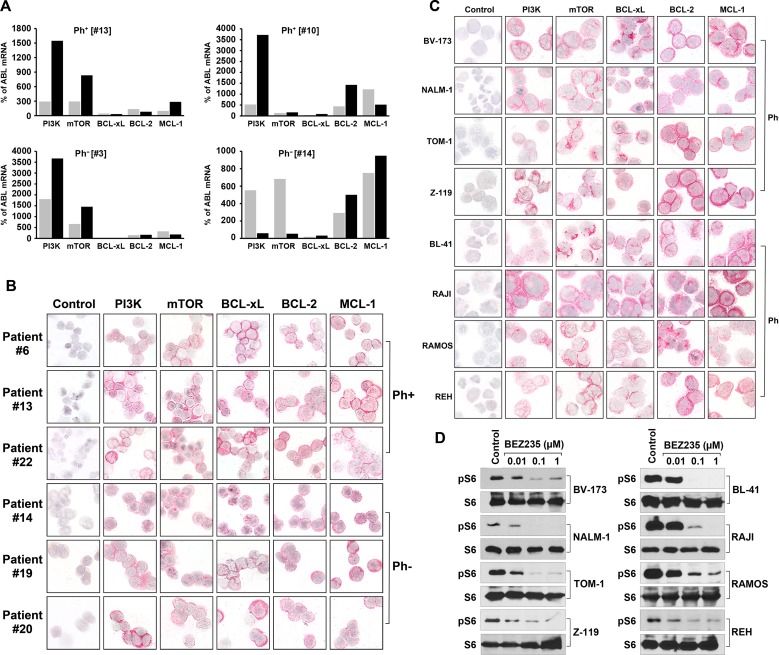
Expression of molecular targets in ALL cells **(A)** Expression of mRNA specific for *PI3-kinase* (PI3K), *mTOR*, *BCL-xL*, *BCL-2*, and *MCL-1*, in highly purified (FACS-sorted) CD34^+^/CD38^+^ progenitor cells (grey bars) and CD34^+^/CD38^−^ stem cells (black bars) obtained from patients with Ph^+^ ALL (left panels) and with Ph^−^ ALL (right panels). qPCR was performed as described in the text. mRNA levels are expressed as percent of *ABL1* mRNA levels. **(B)** Expression of PI3K, mTOR, BCL-xL, BCL-2, and MCL-1 protein in primary, patient-derived ALL cells (3 patients with Ph^+^ ALL, #6, #13, #22; 3 patients with Ph^−^ ALL:#14, #19, #20; numbers refer to identification numbers shown in Table 4 and Supplementary Table 4). Antigen expression was determined by specific antibodies and immunocytochemistry on cytospin slides. **(C)** Detection of drug targets in lymphoid cell lines, including the Ph^+^ ALL cell lines BV173, NALM-1, TOM-1, and Z119, and the Ph^−^ cell lines BL-41, RAJI, RAMOS, REH. Cells were stained with monoclonal antibodies by indirect immunocytochemistry. The antibody-omission control is also shown (left panels in **B** and **C**). Technical details are described in the text. **(D)** Ph^+^ ALL cell lines (left panel) and Ph^−^ cell lines (right panel) were incubated in control medium or in various concentrations of BEZ235 as indicated at 37°C for 4 hours. Then, cells were lysed and Western blotting was performed using antibodies against S6 or phosphorylated S6 (pS6).

As assessed by qPCR, primary mononuclear cells of all patients with Ph^+^ ALL (n=3) and Ph^−^ ALL (n=5) tested were found to express transcripts for *PI3-kinase*, *mTOR*, *BCL-xL*, *BCL-2* and *MCL-1* (Table 1). We were also able to demonstrate that primary CD34^+^/CD38^−^ cell populations, known to contain leukemic stem cells (LSC), express *PI3-kinase*, *mTOR*, *BCL-xL*, *BCL-2*, and *MCL-1* mRNA (Figure 1A, Table 1). In most patients, ALL cells expressed lower amounts of *BCL-xL* mRNA compared to the other BCL-2 family members tested (Figure 1A, Table 1). All lymphoid cell lines examined were found to express transcripts for *PI3-kinase*, *mTOR*, *BCL-xL*, *BCL-2*, and *MCL-1* (Table 2). Again, ALL cell lines expressed lower amounts of *BCL-xL* mRNA compared to other *BCL-2* family members (Table 2 and Supplementary Table 1). As assessed by Western blotting, all cell lines were found to express these targets at the protein level (Supplementary Table 2 and Supplementary Figure 1). We also confirmed expression of these growth- and survival regulators in primary ALL cells (Figure 1B) and in all cell lines by immunocytochemistry (Figure 1C). In antibody-dilution experiments, the Ph^+^ cell lines NALM-1, TOM-1, and Z119 were found to express lower levels of BCL-xL and MCL-1 compared to the Ph^−^ cell lines BL41, RAJI, and RAMOS (Supplementary Table 3). Pre-incubation of the anti-BCL-xL antibody with a specific blocking peptide resulted in a negative stain (Supplementary Figure 2). In a next step, we confirmed that the PI3-kinase/mTOR pathway is activated in ALL cell lines by Western blotting using an antibody against phosphorylated (p) S6 (pS6) (Figure 1D). Expression of pS6 in these cell lines was also confirmed by flow cytometry (Supplementary Figure 3). These data suggest that ALL cells express components of the PI3-kinase/mTOR pathway as well as multiple BCL-2 family members. As expected, BEZ235 was found to downregulate the expression of pS6 in all ALL cell lines tested (Figure 1D and Supplementary Figure 3).

**Table 1:** Expression of molecular targets (mRNA level) in primary ALL cells

Patient #		PI3K	mTOR	BCL-xL	BCL-2	MCL-1
2	MNC	+++	++	+	+	+
2	CD34+/CD38+	n.t.	n.t.	n.t.	n.t.	n.t.
2	CD34+/CD38-	n.t.	n.t.	n.t.	n.t.	n.t.
3	MNC	n.t.	n.t.	n.t.	n.t.	n.t.
3	CD34+/CD38+	+++	+++	+	++	++
3	CD34+/CD38-	+++	+++	+	++	++
6	MNC	+++	++	+	+++	+++
6	CD34+/CD38+	n.t.	n.t.	n.t.	n.t.	n.t.
6	CD34+/CD38-	+++	++	+	++	++
7	MNC	n.t.	n.t.	+	++	+++
7	CD34+/CD38+	n.t.	n.t.	+++	++	+++
7	CD34+/CD38-	n.t.	n.t.	++	++	+++
10	MNC	+++	++	++	+++	+++
10	CD34+/CD38+	+++	++	+	++	+++
10	CD34+/CD38-	+++	++	+	+++	+++
13	MNC	+	+	+	+	++
13	CD34+/CD38+	++	++	+	++	+
13	CD34+/CD38-	+++	+++	+	+	++
14	MNC	+	+	+	+	+
14	CD34+/CD38+	+++	+++	+	++	+++
14	CD34+/CD38-	+	+	+	++	+++
15	MNC	n.t.	n.t.	n.t.	n.t.	n.t.
15	CD34+/CD38+	++	+	+	+	+
15	CD34+/CD38-	+	++	+	+	+
17	MNC	n.t.	n.t.	n.t.	n.t.	n.t.
17	CD34+/CD38+	++	++	+	++	++
17	CD34+/CD38-	n.t.	++	+	+	++
18	MNC	+++	++	++	+++	++
18	CD34+/CD38+	+++	++	++	+++	++
18	CD34+/CD38-	+++	+++	++	+++	+++
20	MNC	+++	+++	+	+++	+++
20	CD34+/CD38+	++	+++	+	+++	+++
20	CD34+/CD38-	n.t.	n.t.	n.t.	n.t.	n.t.

Unfractionated mononuclear cells (MNC) and highly purified (sorted) leukemic stem and progenitor cells (CD34+/CD38- and CD34+/CD38+ cells) were subjected to RNA isolation and analyzed for expression of mRNAs specific for PI3 kinase (PI3K), the mechanistic target of rapamycin (mTOR), BCL-xL, BCL-2, and MCL-1 by qPCR as described in the text. Transcript levels were calculated as percent of ABL1 mRNA and shown using the following score: +, ≤100% (of *ABL1*), ++, 100-500%, +++, >500%. n.t., not tested.

**Table 2:** Expression of BCL-2 family members (mRNA level) in lymphoid cell lines

	PI3K	mTOR	BCL-xL	BCL-2	MCL-1
BV-173	++	++	+	++	+
NALM-1	++	++	+	++	+
TOM-1	++	++	+	+	+++
Z-119	++	++	+	++	++
BL-41	+++	++	+	++	+++
RAJI	++	+++	+	+++	+++
RAMOS	+++	+++	++	+	+++
REH	++	++	+	++	+

Cell lines were examined for expression of mRNAs specific for the above listed targets by qPCR as described in the text. Transcript levels were calculated as percent of *ABL1* mRNA. The table shows the mRNA expression levels using the following score:

+, ≤100% (of *ABL1*), ++, 100-500%, +++, >500%.

### Effects of obatoclax and BEZ235 on growth of ALL cells

**Figure 2:**
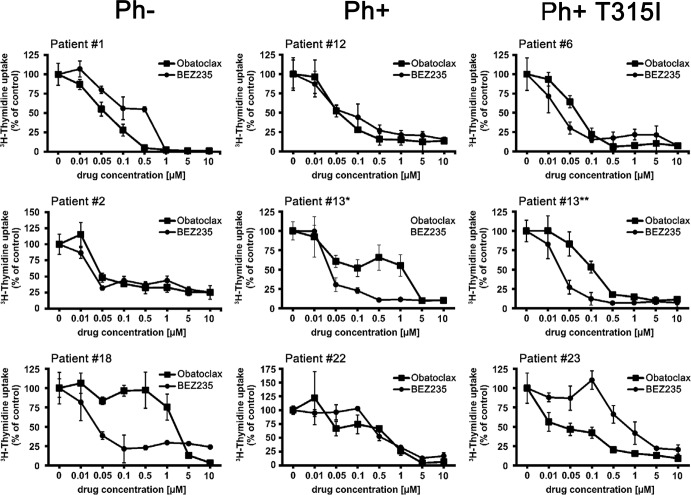
Effects of obatoclax and BEZ235 on proliferation of primary ALL cells Primary leukemic blast cells obtained from patients with Ph^−^ ALL (left panels), Ph^+^ ALL (middle panels), and Ph^+^ ALL exhibiting BCR-ABL1 T315I (right panels) were incubated in control medium or medium containing various concentrations of BEZ235 or obatoclax (as indicated) at 37°C for 48 hours. Then, ^3^H-thymidine uptake was measured as described in the text. Results are expressed as percent of control and represent the mean±S.D. of triplicates. Patient numbers (#) refer to identification-numbers shown in Table 4 and Supplementary Table 4. In patient #13, blast cells were examined at the time of diagnosis (*) where no BCR-ABL1 mutation was detected and at the time of relapse when lymphoblastic cells were found to display BCR-ABL1 T315I (**).

Obatoclax was found to inhibit the proliferation of all Ph^+^ ALL cell lines and all Ph^−^ cell lines tested (Table 3). The effects of obatoclax were dose-dependent, with IC_50_ values ranging between 0.05 and 0.5 μM in the Ph^+^ cell lines employed, and between 0.1 and 0.3 μM in the Ph^−^ cell lines tested (Table 3). Similarly, BEZ235 was found to suppress proliferation in all lymphoblastic cell lines examined, with IC_50_ values ranging between 0.01 and 0.08 μM (Table 3). In a next step we examined drug effects on primary ALL cells. In these experiments, obatoclax and BEZ235 were found to inhibit the proliferation of primary ALL cells in all patients tested, with IC_50_ values ranging between 0.01 and 5.0 μM for obatoclax and between 0.01 μM and 1.0 μM for BEZ235 (Figure 2, Table 4). We were also able to show that BEZ235 downregulates the expression of pS6 in all cell lines tested (Figure 1D and Supplementary Figure 3). By contrast, no substantial effects on growth of ALL cells were seen with the selective mTOR inhibitor everolimus (IC_50_ >1 μM; not shown). In control experiments, BEZ235 did not exert substantial effects on proliferation of normal BM precursor cells whereas obatoclax suppressed the proliferation of these cells (IC_50_ 0.1-0.5 μM). Next, we were interested to learn whether obatoclax and BEZ235 would also block proliferation in leukemic cells exhibiting various imatinib-resistant mutant forms of BCR-ABL1. We found that both drugs inhibit the proliferation of Ba/F3 cells expressing ‘wild type’ (wt) *BCR-ABL1* or various *BCR-ABL1* mutants, including T315I, with reasonable IC_50_ values (Supplementary Figure 4 and Supplementary Table 4). Finally, we were able to show that obatoclax and BEZ235 inhibit the growth of primary ALL cells derived from patients exhibiting the T315I mutant of BCR-ABL1 (Figure 2 and Table 4). These data suggest that obatoclax and BEZ235 exert substantial anti-neoplastic effects on leukemic cells in ALL, including TKI-resistant cells.

**Table 3:** Response of lymphoblastic cell lines to obatoclax and BEZ235

Cell Line	Diagnosis	*BCR-ABL1*	Proliferation	
			Obatoclax IC_50_ [μM]	BEZ235 IC_50_ [μM]
BV-173	B-ALL	p210	0.1-0.3	0.01-0.03
NALM-1	CML, lymphoid BP	p210	0.1-0.5	0.05-0.08
TOM-1	B-ALL	p190	0.1-0.3	0.01-0.03
Z-119	B-ALL	p190	0.05-0.08	0.03-0.05
BL-41	Burkitt lymphoma	-	0.1-0.15	0.01-0.03
RAJI	Burkitt lymphoma	-	0.1-0.3	0.05-0.08
RAMOS	Burkitt lymphoma	-	0.1-0.3	0.03-0.05
REH	B-ALL	-	0.1-0.3	0.03-0.05

Cell lines were cultured in control medium or in various concentrations of obatoclax or BEZ235 for 48 hours. Thereafter, proliferation was measured by ^3^H-thymidine incorporation assay. IC_50_ values (μM) represent the ranges from at least 3 independent experiments. Abbreviations: CML, chronic myeloid leukemia; BP, blast phase; ALL, acute lymphoblastic leukemia; p210, BCR-ABL1 major breakpoint; p190, BCR-ABL1 minor breakpoint.

**Table 4:** Effects of obatoclax and BEZ235 on proliferation of primary ALL cells

Patient No. (#)	Proliferation	
	obatoclax	BEZ235
	**IC_50_ [μM]**	**IC_50_ [μM]**
1	0.05-0.1	0.5-1
2	0.01-0.05	0.01-0.05
4	0.01-0.1	0.01-0.1
6*	0.05-0.1	0.01-0.05
7	0.5-1	0.05-0.1
8	0.01-0.1	0.1-0.5
9	0.5-1	0.5-1
11	1-5	0.1-0.5
12	0.05-0.1	0.05-0.1
13	1-5	0.01-0.05
13*	0.05-0.1	0.01-0.05
13*	0.1-0.5	0.01-0.05
18	1-5	0.01-0.05
21	0.1-0.5	0.05-0.1
22	0.5-1	0.5-1
23*	0.01-0.05	0.5-1

Primary ALL cells were cultured in control medium or in various concentrations of obatoclax or BEZ235 for 48 hours. Thereafter, ^3^H-thymidine-uptake was determined. IC_50_ values (μM) obtained in each patient are shown. *In these investigations, leukemic cells expressed BCR-ABL1 T315I at the time of drug testing.

### Drug effects on cell cycle progression and apoptosis in ALL cells

**Figure 3:**
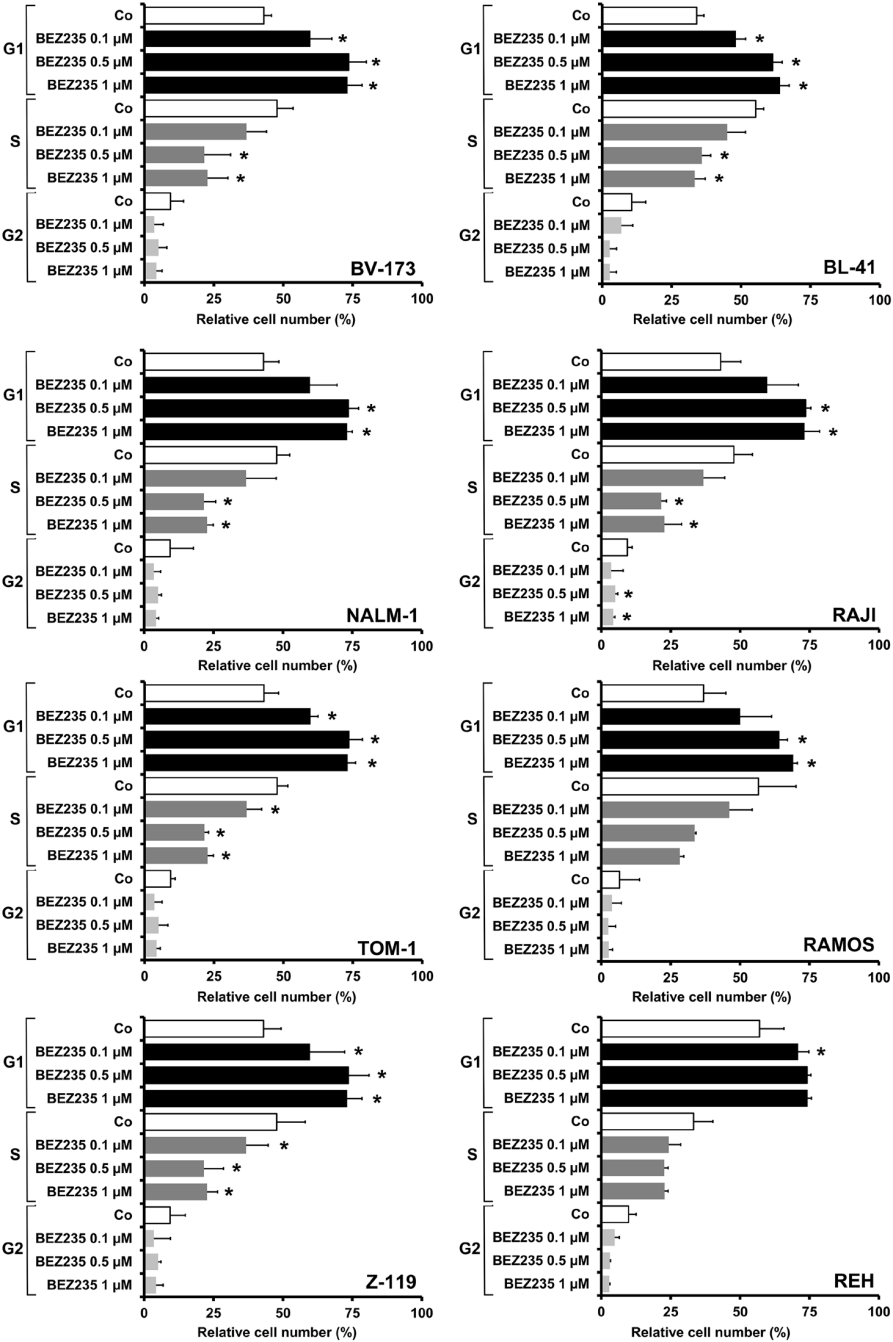
Drug effects on cell cycle progression in ALL cell lines Ph^+^ cell lines (left panel: BV173, NALM-1, TOM-1, Z119) and Ph^−^ cell lines (right panel: BL-41, RAJI, RAMOS, REH) were incubated in control medium (Co) or in various concentrations of BEZ235, as indicated, at 37°C for 48 hours. Then, cell cycle progression was analyzed by flow cytometry as described in the text. Results are expressed as percent of control and represent the mean±S.D. of three independent experiments. Asterisk (*): p<0.05 compared to control.

**Figure 4:**
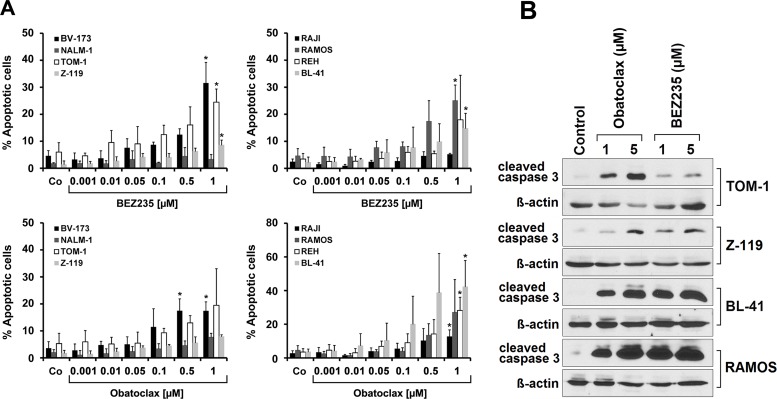
Drug effects on apoptosis in ALL cells **(A)** Lymphoid cell lines were incubated in control medium (Co) or in various concentrations of BEZ235 (upper panels) or obatoclax (lower panels) as indicated at 37°C for 48 hours. Then, cells were harvested and examined for the numbers (percentage) of apoptotic cells by light microscopy. Results are expressed as percent of apoptotic cells and represent the mean±S.D. of at least three independent experiments. Asterisk (*): p<0.05 compared to control. **(B)** Western blot experiment was performed with TOM-1, Z-119, BL-41, and RAMOS using antibodies against cleaved caspase 3 or Δ-Actin. Cells were incubated in control medium or in the presence of obatoclax (1 or 5 μM) or BEZ235 (1 or 5 μM) at 37°C for 24 hours. Western blotting was performed as described in the text. Δ-Actin served as a loading control.

In order to study the mechanism of drug-induced growth inhibition, we performed cell cycle studies in drug-exposed cells. As assessed by flow cytometry BEZ235 was found to induce a G1 cell cycle arrest in all Ph^+^ and all Ph^−^ cell lines tested (Figure 3). Because of its fluorescence, we were not able to perform cell cycle experiments and other flow cytometry experiments with obatoclax. In a next step we examined the effects of obatoclax and BEZ235 on survival and signs of apoptosis in ALL cells. In these experiments, we were able to show that both drugs increase the number of apoptotic cells, detected by microscopy, in all Ph^+^ and Ph^−^ cell lines examined (Figure 4A). We also confirmed drug-induced apoptosis by Western blotting using an antibody against cleaved caspase 3 (Figure 4B). In addition, the BEZ235-induced apoptosis was demonstrable by staining cell lines for Annexin V/PI and for active caspase 3 by flow cytometry (Supplementary Figure 5).

### Obatoclax and BEZ235 synergize in producing growth inhibition in ALL cells

**Figure 5:**
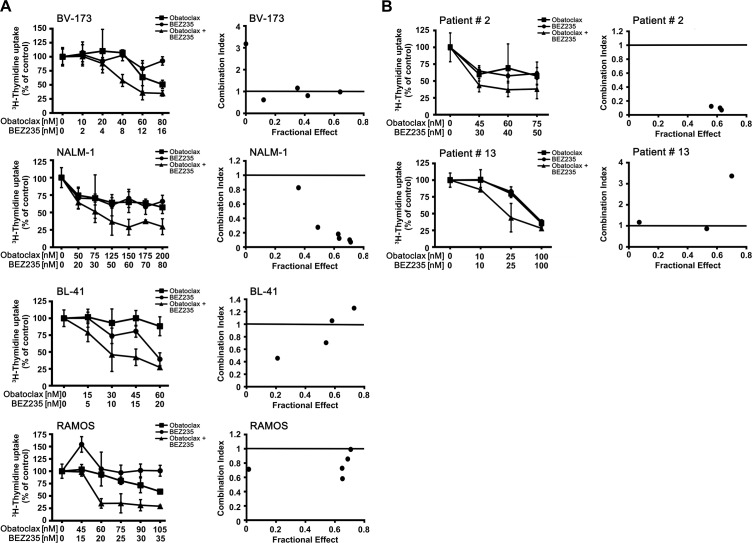
Drug combination effects **(A)** The Ph^+^ ALL cell lines BV-173 and NALM-1 and the Ph^−^ cell lines BL-41 and RAMOS were incubated in control medium (0), in medium containing various concentrations of obatoclax or BEZ235, or in a combination of both drugs at fixed ratio of drug-concentrations (as indicated) at 37°C for 48 hours. After incubation, uptake of ^3^H-thymidine was measured. Results are expressed as percent of control and represent the mean±SD of triplicates (left panels). In the right panels, combination index (CI) values, calculated from fractional effects by Calcusyn software, are shown. A CI value of 1 indicates an additive effect, and CI values below 1 are indicative of synergistic drug effects. **(B)** Primary ALL cells obtained from a patient with Ph- ALL (#2) and one ALL patient with Ph+ ALL (#13) were incubated in control medium (0), in medium containing various concentrations of obatoclax or BEZ235, or in a combination of both drugs at fixed ratio of drug-concentrations (as indicated) at 37°C for 48 hours. After incubation, uptake of ^3^H-thymidine was measured. Results are expressed as percent of control and represent the mean±SD of triplicates.

Whereas obatoclax is a well-established pan-inhibitor of anti-apoptotic members of the BCL-2 family, BEZ235 acts on a completely different type of target, namely the PI3-kinase/mTOR pathway. Therefore, it was reasonable to suggest that these two drugs produce cooperative anti-leukemic effects. To test this hypothesis, we performed ^3^H-thymidine uptake experiments. We found that obatoclax and BEZ235 synergize with each other in producing growth-inhibition in the Ph^+^ cell lines BV-173, NALM-1 and Z-119 and in the Ph^−^ cell lines BL-41 and RAMOS (Figure 5A, Table 5). We also examined whether obatoclax or BEZ235 and various BCR-ABL1 TKI would exert synergistic antineoplastic effects on ALL cells. In most cell lines, these combinations did not produce synergistic effects. However, in the Ph+ cell line Z-119, the drug combinations ‘nilotinib+obatoclax’, ‘imatinib+obatoclax’, and ‘nilotinib+BEZ235’ produced synergistic growth-inhibitory effects (Table 5). Finally, we examined drug combination effects in primary ALL cells. In two patients with ALL (Ph- ALL [#2], Ph+ ALL [#13]), clear cooperative and in part synergistic effects of the drug combination ‘obatoclax+ BEZ235’ on growth of ALL blasts could be demonstrated (Figure 5B). However, in 2 other ALL patients (Ph- ALL [#18]; Ph+ ALL [#23]), no synergistic drug effects were obtained (Supplementary Figure 6). Synergistic drug effects were confirmed by calculating combination index values using Calcusyn software.

**Table 5:** Cooperative anti-proliferative effects of various drug combinations in lymphoid cell lines

	BV-173	NALM-1	TOM-1	Z-119	BL-41	RAJI	RAMOS	REH
Obatoclax + BEZ235	S	S	-	S	S	-	S	-
Obatoclax + Nilotinib	A	-	-	S	n.t.	n.t.	n.t.	n.t.
Obatoclax + Imatinib	-	-	S	S	n.t.	n.t.	n.t.	n.t.
BEZ235 + Nilotinib	-	S	-	S	n.t.	n.t.	n.t.	n.t.
BEZ235 + Imatinib	-	-	-	A	n.t.	n.t.	n.t.	n.t.

Lymphoid cell lines were treated with various concentrations of obatoclax, BEZ235, nilotinib, imatinib or with combinations of these drugs. Anti-proliferative effects were measured by ^3^H-thymidine uptake. The combination index (CI) was determined by Calcusyn software. Synergistic (S) effects are defined by a CI <1; additive (A) effects by a CI=1; and less than additive effects by a CI >1 (‘-’). n.t., not tested.

## DISCUSSION

Recent data suggest that components of the PI3-kinase/mTOR pathway and members of the BCL-2 family are important triggers of growth and survival in lymphoid blast cells in ALL [22–25]. It has also been described that drugs directed against these therapeutic targets can counteract growth and survival of Ph^+^ and Ph^−^ ALL cells [26, 27, 30–32]. In the present study we have extended these analyses by demonstrating cytoreductive, cell cycle-targeting, and apoptosis-inducing effects of two drugs, the dual PI3-kinase and mTOR blocker BEZ235, and obatoclax, a drug directed against a number of pro-survival members of the BCL-2 family. Moreover, our data show that combined targeting of the PI3-kinase/mTOR pathway and BCL-2 family members using BEZ235 and obatoclax leads to synergistic growth-inhibitory effects in ALL cells.

Expression of PI3-kinase, mTOR, and members of the BCL-2 family in ALL cells was demonstrable by qPCR, Western blotting, and immunocytochemistry. Ph^+^ ALL cells as well as Ph^−^ ALL cells were found to express these target molecules, suggesting that apart from *BCR-ABL1*, other mechanisms and pathways also contribute to expression of these survival molecules in ALL cells. The baseline levels of all targets examined were largely comparable in the Ph^+^ and Ph^−^ cell lines tested. However, higher levels of BCL-2 family members were detected in the Ph^−^ cell lines, with the exception of low level expression of BCL-2 in RAMOS cells. Similar results were obtained with PI3-kinase and mTOR. These data argues against a particular role of *BCR-ABL1* in expression of these target antigens in lymphoblasts. Rather, we believe that several different pathways and pro-oncogenic molecules contribute to expression of PI3-kinase, mTOR, and BCL-2 family members in lymphoid leukemias.

Recent data suggest that drugs targeting various components of the PI3-kinase/mTOR pathway or members of the BCL-2 family in ALL cells can induce growth inhibition [26, 27, 30–32]. In the current study, we confirmed these data and found that BEZ235 and obatoclax induce dose-dependent growth inhibition and apoptosis in all Ph^+^ and all Ph^−^ cell lines tested. Moreover, both drugs were found to inhibit the growth of primary leukemic cells in all patients with Ph^+^ ALL and all patients with Ph^−^ ALL tested. These data suggest that inhibition of the PI3-kinase/mTOR pathway and of anti-apoptotic members of the BCL-2 family may be a reasonable approach to counteract malignant cell growth in ALL. In normal BM cells BEZ235 did not produce substantial growth-inhibitory effects, whereas obatoclax was found to inhibit the proliferation of normal BM cells. We also examined whether mTOR plays a particular role as a drug target in ALL cells. To address this question, we applied the mTOR-specific inhibitor everolimus. However, in contrast to BEZ235, everolimus failed to inhibit the proliferation of ALL cells at pharmacologically meaningful drug concentrations (IC_50_ >1 μM). Collectively, these data suggest that PI3-kinase and other PI3-kinase-downstream molecules (other than mTOR) play a more important role as targets of BEZ235 compared to mTOR.

Several different members of the BCL-2 family have been implicated in the regulation of growth and survival of ALL cells [30–32]. In the present study, we were able to show that Ph^+^ and Ph^−^ ALL cells, including the CD34^+^/CD38^−^ stem cells and the CD34^+^/CD38^+^ progenitor cells, express BCL-2, BCL-xL, and MCL-1 in a constitutive manner. Correspondingly, the pan BCL-2 blocker obatoclax was found to induce growth arrest and apoptosis in ALL cells. So far, it remains unknown which BCL-2 family members were responsible for these drug effects. In fact, several different BCL-2 family members may act together to contribute to growth and survival of ALL cells, and thus combined targeting of these molecules may be required to obtain major drug effects [30, 31].

Drug resistance is a major challenge in patients with advanced ALL [34–37]. In many patients with drug-resistant Ph^+^ ALL, TKI-resistant mutant forms of BCR-ABL1 are detected [36–39]. The most frequent and clinically relevant mutant of BCR-ABL1 is T315I [16, 17, 40]. In order to define the effects of obatoclax and BEZ235 on growth of *BCR-ABL1*-mutated leukemic cells, Ba/F3 cells expressing various BCR-ABL1 mutations were employed. We found that both drugs inhibit the growth of Ba/F3 cells expressing diverse BCR-ABL1 mutants, including T315I. In addition, we were able to show that BEZ235 and obatoclax inhibit the proliferation of primary ALL cells obtained from patients with TKI-resistant ALL, including cases with BCR-ABL1 T315I. This observation may have clinical implications, as TKI resistance remains a major therapeutic challenge in Ph^+^ ALL.

While targeted therapies like TKI have untoward effects of their own, they are relatively mild compared to conventional therapy and the lack of additive adverse effects allow for combination therapy with other targeted agents or conventional anti-neoplastic drugs. Nevertheless, obatoclax and BEZ235 may also exert some inhibitory effects on normal healthy cells. An attractive strategy to simultaneously avoid side effects and overcome drug resistance in ALL may be to combine various targeted drugs with each other or with conventional drugs. A number of different studies have shown that both the PI3-kinase/mTOR pathway and members of the BCL-2 family play a role in growth and survival of ALL cells [22–30]. However, although a cross-talk in the signalling cascades may sometimes be present, the two target pathways tested (PI3-kinase/mTOR and BCL-2-dependent signalling) are considered to be activated and to trigger oncogenesis independent of each other. Therefore, it was reasonable to assume that the two drugs applied may produce cooperative or even synergistic anti-leukemic effects. In addition, in the Ph+ ALL cell lines, we combined these drugs with BCR-ABL TKI. In these experiments we were indeed able to demonstrate cooperative anti-leukemic drug effects. In particular, in 5 out of the 8 lymphoid cell lines examined, BEZ235 and obatoclax were found to produce synergistic effects on proliferation. Moreover, in the Ph^+^ cell line Z-119, both drugs were found to cooperate with the BCR-ABL1 TKI imatinib and nilotinib in suppressing cell proliferation.

Together, our data show that PI3-kinase and members of the BCL-2 family are important target antigens in Ph^+^ and Ph^−^ ALL cells, including lymphoblastic cells that have acquired resistance against imatinib. Moreover, our data also show that combined targeting of the PI3-kinase/mTOR pathway and a broader spectrum of BCL-2 family members is associated with reduced proliferation and survival in ALL cells. Whether this concept can be translated into clinical practice remains to be determined in consecutive preclinical investigations and clinical trials. Since several inhibitors of BCL-2 family members, like venetoclax, and PI3-kinase blockers, are available and are already used in clinical trials in patients with lymphoproliferative disorders, the development of combined targeting approaches may be straightforward and promising.

## MATERIALS AND METHODS

### Reagents

Imatinib, nilotinib (AMN107), and BEZ235 were kindly provided by Dr. E. Buchdunger and Dr. P. Manley (Novartis Pharma AG, Basel, Switzerland). Ponatinib and RAD001 (everolimus) were purchased from SelleckChem (Houston, TX, USA) and GX015-070 (obatoclax) from ChemieTek (Indianapolis, IN, USA). Stock solutions of drugs were prepared by dissolving in dimethyl-sulfoxid from Sigma-Aldrich (St. Louis, MO, USA). RPMI 1640 medium was purchased from Lonza (Verviers, Belgium), fetal bovine serum (FBS) from Thermo Fisher Scientific (Waltham, MA, USA), and bovine serum albumin (BSA) from Sigma-Aldrich. Propidium Iodide (PI) was purchased from Sigma-Aldrich, Annexin V from Affymetrix (Santa Clara, CA, USA), and ^3^H-thymidine from Perkin Elmer (Waltham, MA, USA). A characterization of polyclonal and monoclonal antibodies (mAb) used in this study is provided in Supplementary Table 5.

### Primary ALL cells and cell lines

For *in vitro* culture experiments, primary leukemic cells were obtained from 23 patients with Ph^+^ ALL (n=9) and Ph^−^ ALL (n=14), including patients with common ALL (c-ALL, n=13), pre B-ALL (n=5), pro B-ALL (n=1), T-ALL (n=2), and mixed phenotypic ALL (n=2). Heparinized BM and peripheral blood (PB) was layered over Ficoll to isolate mononuclear cells (MNC). For polymerase chain reaction (PCR) analysis, frozen samples from 5 patients with Ph^+^ ALL and 6 with Ph^−^ ALL were used. In 9 patients (5 with Ph^+^ ALL and 4 with Ph^−^ ALL), CD34^+^/CD38^−^ cells and CD34^+^/CD38^+^ cells were purified by cell sorting (purity >98%) as described [28, 41]. The patients' characteristics are shown in Supplementary Table 6. Written informed consent was obtained in each case. The study was approved by the ethics committee of the Medical University of Vienna, Austria. The Ph^+^ cell lines BV-173, TOM-1, NALM-1, and Z-119, and the Ph^−^ lymphatic cell lines RAJI, RAMOS, REH, and BL-41 were used. Z-119 cells were kindly provided to J.V.M. by Dr. Zeev Estrov (MD Anderson Cancer Centre, Houston, Texas, USA). All other human cell lines were purchased from the Leibniz Institute DSMZ-German Collection of Microorganisms and Cell Cultures (Braunschweig, Germany). The identity of the cell lines was confirmed by DNA sequencing and DNA profiling (by nonaplex PCR) and by studying the presence or absence of *BCR-ABL1*. Cell lines were cultured in RPMI 1640 medium and 20% heat-inactivated FCS at 37°C and 5% CO_2_. Table 3 shows a summary of human lymphoid cell lines tested in this study. In a separate set of experiments, Ba/F3 cells expressing wild type (wt) human *BCR-ABL1* or various *BCR-ABL1* mutations (Q252H, M244V, E255V, G250E, F359V, F317L, T315I, Y253H, H396P, F317V, E255K) were tested. These cell lines were kindly provided by Dr. Michael Deininger (Salt Lake City, UT, USA). Ba/F3 clones were cultured in RPMI 1640 medium containing 10% FCS.

### Quantitative PCR (qPCR)

RNA was isolated from primary ALL cells and cell lines using the RNeasy MinEluteCleanupKit (Qiagen, Hilden, Germany). cDNA was synthesized using Moloney murine leukemia virus reverse transcriptase (Invitrogen, Carlsbad, CA, USA), random primers, first strand buffer, dNTPs (100 mM), and RNasin (all from Invitrogen) according to the manufacturer's instructions. PCR was performed as reported [39, 42] using primers specific for PI3K, mTOR, BCL-xL, BCL-2, MCL-1, Δ-Actin and ABL1 (Supplementary Table 7). mRNA levels were quantified on a 7900HT Fast Real-Time PCR System (Applied Biosystem, Foster City, CA, USA) using iTAq SYBR Green Supermix with ROX (Bio-Rad, Hercules, CA, USA). *PI3-kinase-*, *mTOR-*, *BCL-xL*-, *BCL-2-*, and *MCL-1* mRNA expression levels were normalized to *ABL1* or *Δ-Actin* mRNA levels and expressed as percentage of *ABL1* or *Δ-Actin* mRNA. Calculations were based on standard curves established for *PI3-kinase*, *mTOR*, *BCL-xL*, *BCL-2*, *MCL-1*, *ABL1*, and *Δ-Actin* mRNA expression.

### Western blotting

Cells were incubated in control medium or in medium containing obatoclax (1 to 5 μM) or BEZ235 (0.01 to 5 μM) for 4 or 48 hours. Then Western blotting was performed essentially as described [42] using the primary antibodies listed in Supplementary Table 3. Antibody reactivity was made visible with a donkey anti-rabbit IgG and Pierce ECL Plus Western blotting substrate (Thermo Fisher Scientific).

### Immunocytochemistry

Immunocytochemistry was performed on cytospin-slides prepared with primary neoplastic cells and cell lines as described [28]. Slides were incubated with primary antibodies against PI3K, mTOR, BCL-xL, BCL-2, or MCL-1 (Supplementary Table 5) overnight, washed, and then incubated with a biotinylated second-step antibody (Biocare Medical, Concord, CA, USA) for 30 minutes. Streptavidin-alkaline-phosphatase complex (Biocare Medical) was used as chromogen. Antibody reactivity was made visible using Neofuchsin (Nichirei, Tokyo, Japan). Slides were counterstained in Mayer's hemalaun. In a separate set of experiments, various antibody dilutions were applied in order to analyze antigen expression (PI3K, mTOR, BCL-xL, BCL-2, or MCL-1) in a semi-quantitative manner. In control experiments, the BCL-xL antibody was incubated with control buffer or with a BCL-xL-specific blocking peptide (Cell Signaling, Danvers, MA, USA) at room temperature for 30 minutes before being applied in the stain. In control experiments, the anti-BCL-xL antibody was incubated with control buffer or buffer containing a BCL-xL-specific blocking peptide prior to staining.

### Proliferation assay

To examine anti-proliferative effects of targeted drugs, Ph^+^ and Ph^−^ ALL cell lines and primary ALL cells were cultured in 96-well microtiter plates (2 × 10^4^ cells/well) in the absence or presence of various concentrations of BEZ235, obatoclax, ponatinib, nilotinib, imatinib and everolimus for 48 hours. Thereafter, ^3^H-thymidine (0.5 μCi per well) was added for 16 hours. Cells were then harvested on filter membranes (Packard Bioscience, Meriden, CT, USA) in a Filtermate 96 harvester (Packard Bioscience). Filters were air-dried, and the bound radioactivity was measured in a β-counter (Top-Count NXT, Packard Bioscience). All experiments were performed in triplicates. In a separate set of experiments, cell lines were cultured in the presence of various drug combinations (TKI+obatoclax, TKI+BEZ235, and obatoclax+BEZ235) before analyzing ^3^H-thymidine uptake.

### Flow cytometry and cell sorting

In 5 patients with Ph^+^ ALL and in 5 with Ph^−^ ALL, putative ALL stem cells (LSC) were purified to homogeneity (purity 91-98%) by multi-color flow cytometry and cell sorting on a FACSAria (BD Biosciences, San Jose, CA, USA). Purified LSC were checked for cell viability and were then subjected to RNA isolation and qPCR. Cell cycle progression was analyzed by flow cytometry as described [43]. To measure apoptosis in drug-exposed cells, combined AnnexinV/PI staining was performed as described [42, 43]. Because of obatoclax-associated fluorescence, flow cytometry experiments could only be performed with BEZ235-exposed cells. Cells were analyzed by flow cytometry on a FACScan (BD Biosciences). For detection of active caspase-3 and pS6, cell lines were incubated in control medium or in various concentrations of BEZ235 at 37°C for 48 hours (active caspase-3) or 1 hour (pS6). Then, cells were fixed in 2% formaldehyde (room temperature, 10 minutes), permeabilized in 100% methanol at −20°C (15 minutes), washed in PBS plus BSA (0.1%), and then stained with the PE-conjugated mAb C92-605 (BD Biosciences) directed against active caspase-3 or Alexa-647 conjugated mAb N7-548 anti pS6 (BD Biosciences) for 1 hour. Thereafter, cells were analyzed by flow cytometry on a FACSCalibur (BD Biosciences). Apoptosis was also analyzed by conventional microscopy on Wright-Giemsa-stained cytospin slides. Apoptotic cells were recorded using generally accepted morphologic criteria [44]. For cell cycle studies, drug-exposed cells were resuspended in 500 μl permeabilization buffer (0.1% Na-acetate and 0.1% Triton X-100). Then 40 μl PI were added, and cell cycle distribution analyzed on a FACS Calibur.

### Statistical analysis

The paired Student's t test was applied in growth inhibition-experiments. Results were considered significant when p was <0.05. Drug combination effects (additive versus synergistic) were determined by calculating combination index (CI) values using Calcusyn software (Calcusyn; Biosoft, Ferguson, MO, USA) as described [45]. A CI value of 1 indicates additive effects and a CI below 1 indicates synergistic drug interactions.

## SUPPLEMENTARY FIGURES AND TABLES




