# Supplementary material for: The pan-BCL-2-blocker obatoclax (GX15-070) and the PI3-kinase/mTOR-inhibitor BEZ235 produce cooperative growth-inhibitory effects in ALL cells
Source: Oncotarget. 2017 Jun 28;8(40):67709–22. doi: 10.18632/oncotarget.18810 (PMC5620205; doi:10.18632/oncotarget.18810)
Supplement: Supplementary file 1 [file oncotarget-08-67709-s001.pdf]

The pan-BCL-2-blocker obatoclax (GX15-070) and the PI3-kinase/mTOR-inhibitor BEZ235 produce cooperative growth-inhibitory effects in ALL cells

SUPPLEMENTARY MATERIALS

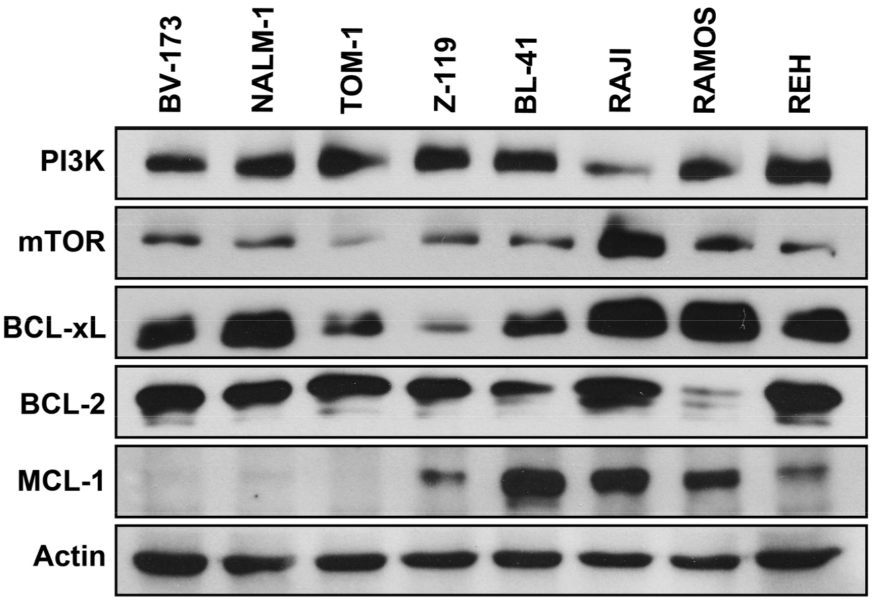

Supplementary Figure 1: Expression of BCL-2 family members in lymphoid cell lines. Ph^+ cells and Ph^- cells lines were lysed and Western Blotting was performed using antibodies against PI3K, mTOR, BCL-xL, BCL-2, and MCL-1. Actin was used as loading control. Technical details are described in the text of the main document.

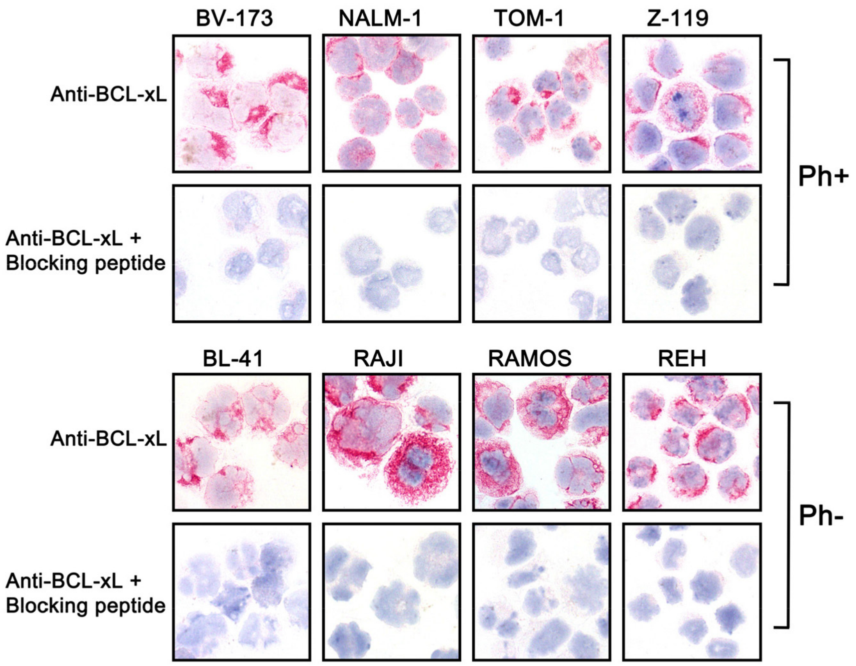

Supplementary Figure 2: Evaluation of specificity of the anti-BCL-xL antibody. Ph^+ cell lines (upper panels) and Ph^- cell lines (lower panels) were spun on cytospin slides and prepared for immunocytochemistry. Prior to staining, the anti-BCL-xL antibody was incubated with a BCL-xL-specific blocking peptide (Anti-BCL-xL + Blocking peptide). Immunocytochemistry was performed as described in the main text of the manuscript. Antibody reactivity was made visible using Neofuchsin.

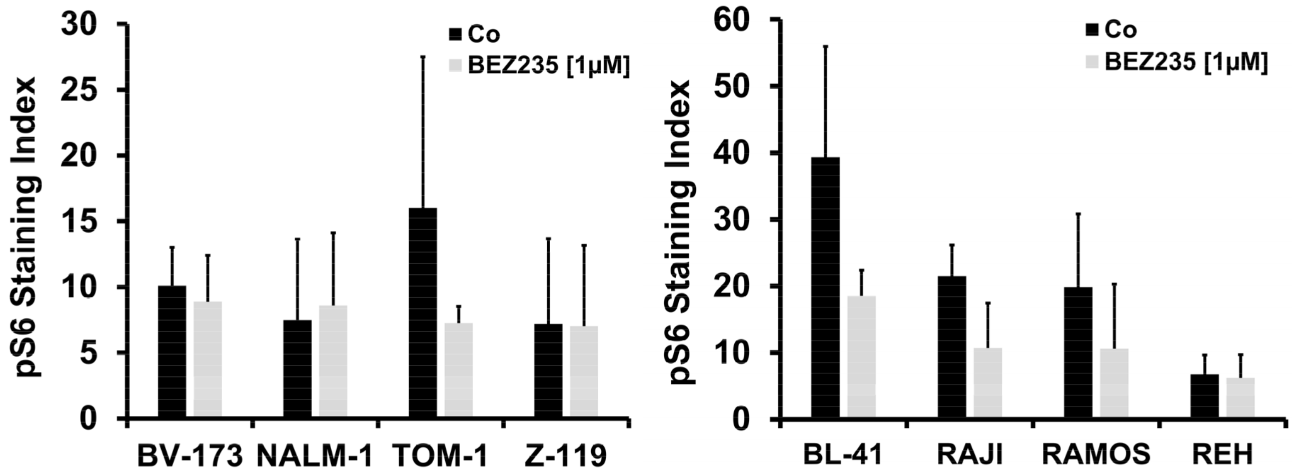

Supplementary Figure 3: Expression of pS6 in various lymphoid cell lines. Cell lines were incubated in control medium (Co; black bars) or BEZ235 (1 µM; grey bars) at 37°C for 1 hour. Then, cells were washed, prepared for cytoplasmic staining (using methanol) and stained with an antibody against pS6 by flow cytometry as described in the text of the main document. Results show the staining index (test antibody relative to an isotype-matched control antibody) and represent the mean±S.D. from three experiments.

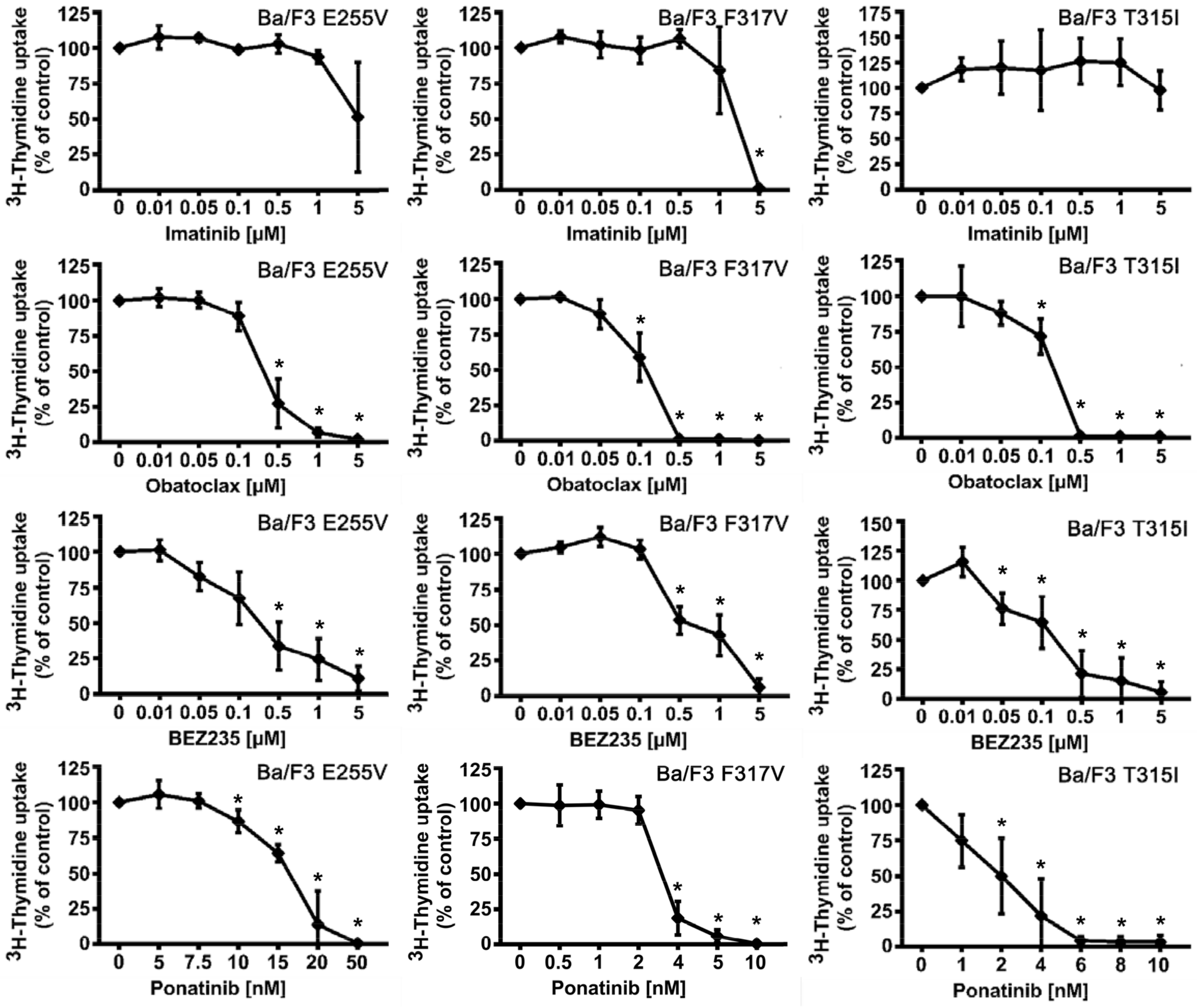

Supplementary Figure 4: BEZ235 and obatoclax inhibit the proliferation of Ba/F3 cells expressing wild type (wt) BCR-ABL1 or various BCR-ABL1 mutants. Effects of imatinib, obatoclax, BEZ235 and ponatinib on proliferation Ba/F3 subclones expressing BCR/ABL mutants: E255V, F317V and T315I. Ba/F3 cells were cultured in RPMI 1640 medium with 10% fetal calf serum (FCS) in the absence (0) or presence of various concentrations (as indicated) of imatinib, obatoclax, BEZ235, or ponatinib at 37°C for 48 hours. Then, uptake of ^{3}H-thymidine was determined. Results are expressed as percent of control and represent the mean±S.D. of three independent experiments. Asterisk (*) indicates: $p<0.05$.

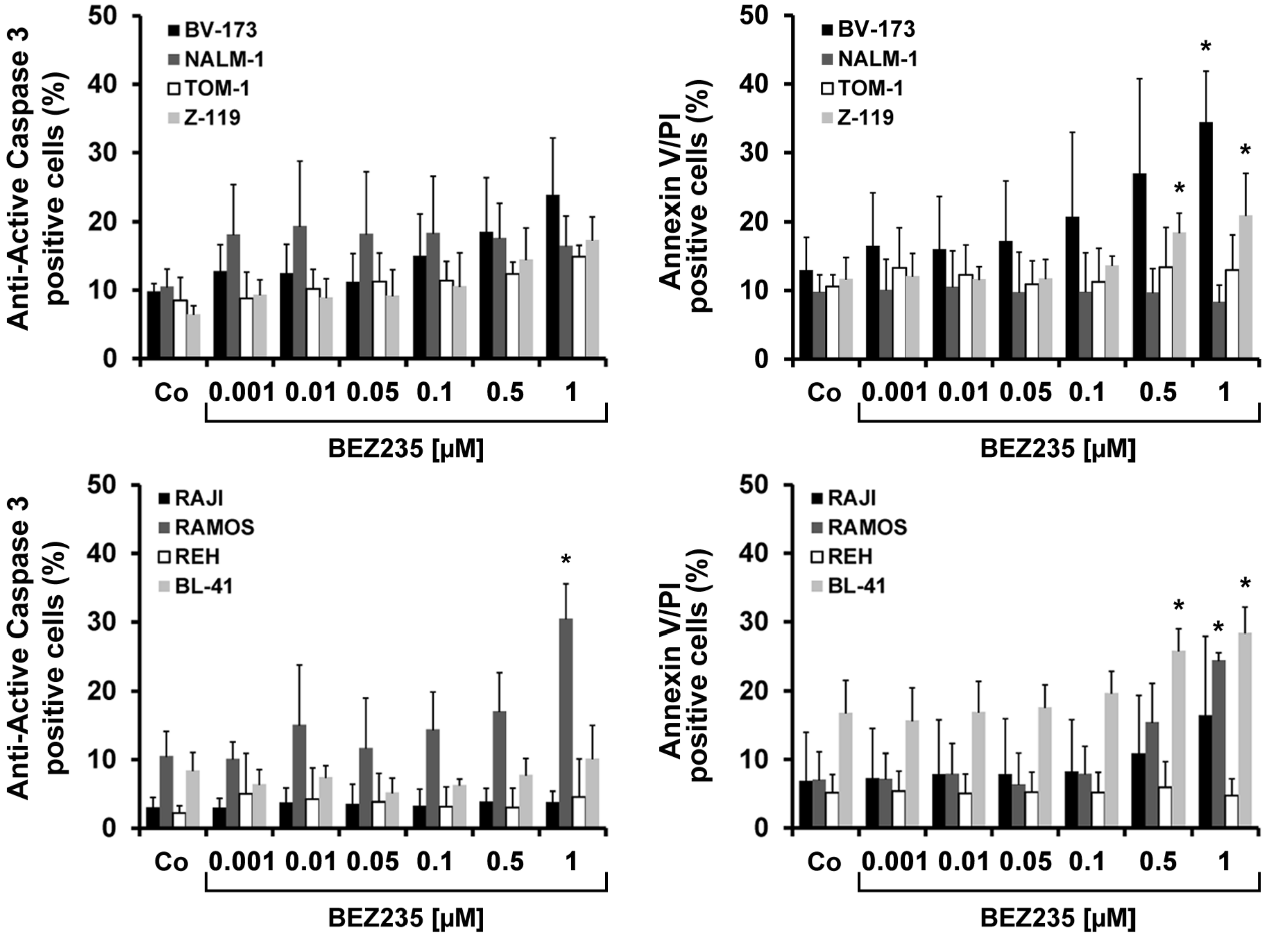

Supplementary Figure 5: BEZ235 induces apoptosis in ALL cells as demonstrated by flow cytometry. Ph^{+} ALL cell lines (upper panels) and Ph^{-} cell lines (lower panels) were incubated in control medium (Co) or in various concentrations of BEZ235 (as indicated) at 37°C for 48 hours. Then, cells were stained with an antibody against active caspase-3 (left panels) or for Annexin V/PI (right panels) by flow cytometry. Technical details are described in the text of the main document. Results show the percentage of active caspase-3-positive cells and Annexin V/PI-positive cells after drug exposure and represent the mean±S.D. of three independent experiments. Asterisk (*) indicates: $p<0.05$ compared to control.

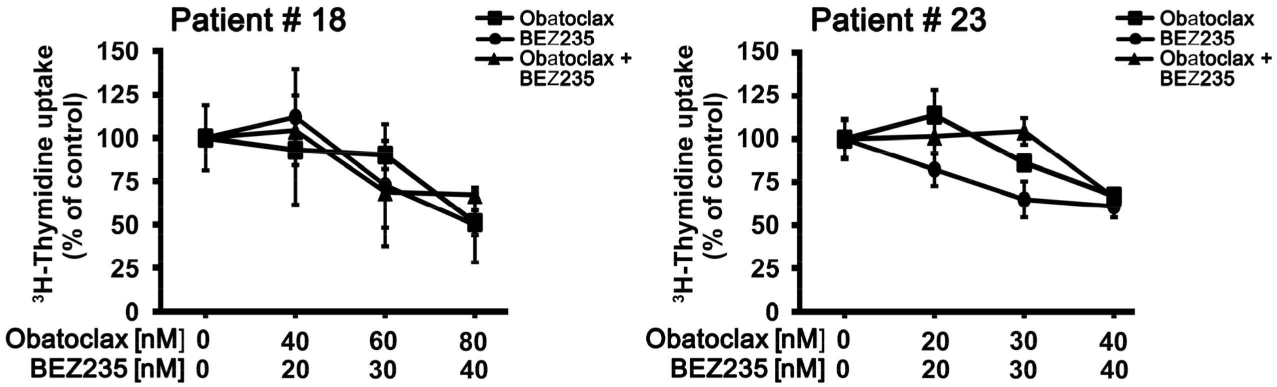

Supplementary Figure 6: Drug combination-effects on proliferation of primary ALL cells. Primary ALL cells obtained from a patient with Ph- ALL (#18; left panel) and one with Ph+ ALL (#23; right panel) were incubated in control medium (0), in medium containing various concentrations of obatoclax or BEZ235, or in a combination of both drugs at fixed ratio of drug-concentrations (as indicated) at 37°C for 48 hours. After incubation, uptake of ^{3}H-thymidine was measured. Results are expressed as percent of control and represent the mean±SD of triplicates. As visible no cooperative drug effects were obtained. However, in two other ALL patients (one with Ph^- ALL and one with Ph^+ ALL), drug combinations resulted in clearly cooperative anti-neoplastic effects (Figure 5B in the main document).

Supplementary Table 1: Expression of BCL-2 family members (mRNA level) in lymphoid cell lines

	PI3K	mTOR	BCL-xL	BCL-2	MCL-1	ABL1
BV-173	++	++	+	++	+	+
NALM-1	++	++	+	++	+	+
TOM-1	+	++	+	+	++	+
Z-119	++	++	+	++	++	+
BL-41	++	++	+	+	++	+
RAJI	+	+++	+	+	++	+
RAMOS	++	+++	+	+	++	+
REH	++	++	+	++	+	++

Cell lines were examined for expression of mRNAs specific for the above listed targets by qPCR as described in the text. Transcript levels were calculated as percent of *ß-Actin* mRNA. The table shows the mRNA expression levels using the following score:

+, ≤0, 5% of *ß-Actin* mRNA;

++, 0, 5-2% of *ß-Actin* mRNA;

+++, >2% of *ß-Actin* mRNA.

Supplementary Table 2: Expression of BCL-2 family members in lymphoid cell lines by Western blotting

Cell line	Antibody against				
	PI3K	mTOR	BCL-xL	BCL-2	MCL-1
BV-173	++	+	++	++	+/-
NALM-1	++	+	++	++	+/-
TOM-1	++	+/-	+	++	-
Z-119	++	+	+/-	++	+
BL-41	++	+	++	+	++
RAJI	+	++	++	++	++
RAMOS	++	+	++	+/-	++
REH	++	+	++	++	+

Expression of BCL-2 family members was determined by Western blotting as described in the text using the following score:

++, strong expression

+, clearly expressed

+/-, weak expression

-, not detectable

Supplementary Table 3: Immunocytochemical evaluation of expression of BCL-2 family proteins

Antibody	Dilution	BV-173	NALM-1	TOM-1	Z-119	BL-41	RAJI	RAMOS	REH
PI3K	1:100	++	+	++	+	+	+	++	+
	1:250	+	+/-	+/-	+/-	+/-	+/-	+	+/-
	1:500	+/-	-	-	+/-	-	+/-	+/-	-
mTOR	1:50	+	++	+	++	+	++	+	++
	1:100	+/-	+	+/-	++	+	++	+	++
	1:250	-	-	+/-	++	+/-	++	+/-	+
BCL-xL	1:100	++	++	++	++	++	++	++	++
	1:250	++	+/-	+	-	++	++	++	+
	1:500	+	-	+/-	-	+	+/-	+	+/-
BCL-2	1:50	++	++	++	++	++	++	++	++
	1:100	++	+	++	++	++	++	++	++
	1:250	+	-	+	+	+	+	+	+
MCL-1	1:25	+	+	+	++	++	++	++	+
	1:50	+/-	+/-	+	+	++	++	++	+
	1:100	+/-	+/-	+	+/-	+	++	++	-

Cell lines were spun on cytospin slides and stained with antibodies at various dilutions as indicated. Technical details are described in the section ‘Materials and Methods’ in the main document. Antibody reactivity was quantified by applying the following score: ++, all cells strongly reactive; +, all cells reactive; +/- weak reactivity or only subsets of cells reactive; -, no reactivity seen.

Supplementary Table 4: Effects of imatinib, obatoclax, BEZ235, and ponatinib on proliferation of Ba/F3 sub-clones carrying various mutant forms of *BCR-ABL1*

Ba/F3 sub-clones *BCR-ABL1* variant	Inhibition of proliferation induced by			
	Imatinib IC_{50} [µM]	Obatoclax IC_{50} [µM]	BEZ235 IC_{50} [µM]	Ponatinib IC_{50} [µM]
wt	0.1-0.5	0.1-0.5	0.5-1	0.001-0.002
Q252H	1-5	0.1-0.5	0.1-0.5	0.001-0.005
M244V	1-5	0.5-1	0.5-1	0.004-0.006
E255V	>5	0.1-0.5	0.1-0.5	0.015-0.02
G250E	1-5	0.1-0.5	0.5-1	0.005-0.01
F359V	1-5	0.1-0.5	0.5-1	0.005-0.0075
F317L	0.5-1	0.1-0.5	0.1-0.5	0.002-0.004
T315I	>5	0.1-0.5	0.1-0.5	0.001-0.004
Y253H	>5	0.1-0.5	0.1-0.5	0.004-0.005
H396P	0.5-1	0.1-0.5	0.5-1	0.001-0.002
F317V	1-5	0.1-0.5	0.5-1	0.002-0.004
E255K	>5	0.1-0.5	0.1-0.5	0.01-0.02

Ba/F3 cells carrying wild type (wt) BCR-ABL1 or various mutant forms of BCR-ABL1 (as indicated) were cultured in control medium or in various concentrations of imatinib, obatoclax, BEZ235, or ponatinib for 48 hours. Then, proliferation was measured by determining ^{3}H-thymidine uptake relative to control medium. IC_{50} values (µM) were calculated from at least 3 independent experiments.

Supplementary Table 5: Characterization of antibodies used in Western blot experiments or immunocytochemistry

Reactive protein	Antibody (clone)	Ig class	Animal source	Provider/ company	Method
PI3 Kinase p110α	C73F8	IgG	Rabbit	Cell Signaling	ICC/WB
mTOR	7C10	IgG	Rabbit	Cell Signaling	ICC/WB
BCL-xL	54H6	IgG	Rabbit	Cell Signaling	ICC/WB
BCL-2 (N-19)	polyclonal	IgG	Rabbit	Santa Cruz	ICC
MCL-1 (S-19)	polyclonal	IgG	Rabbit	Santa Cruz	ICC
MCL-1 (22)	Monoclonal	IgG	Mouse	Santa Cruz	ICC/WB
BCL-2	50E3	IgG	Rabbit	Cell Signaling	WB
Cleaved caspase-3 (Asp175)	polyclonal	n.r	Rabbit	Cell Signaling	WB
AKT	polyclonal	n.r.	Rabbit	Cell Signaling	WB
pAKT (Ser473)	D9E	IgG	Rabbit	Cell Signaling	WB
S6 ribosomal protein	5G10	IgG	Rabbit	Cell Signaling	WB
pS6 ribosomal protein (S235/236)	D57.2.2E	IgG	Rabbit	Cell Signaling	WB
β-Actin (N-21)	polyclonal	IgG	Rabbit	Santa Cruz	WB

Ig, immunoglobulin; ICC, immunocytochemistry; WB, Western blotting; n.r., not reported.

Supplementary Table 6: Patients' characteristics

See Supplementary File 1

Supplementary Table 7: Sequences of primers used in qPCR analyses

Gene name	Primer sequence	
	forward	reverse
**human BCL-2**	TTGACAGAGGATCATGCTGTACTT	TCAGTCTACTTCCTCTGTGATGTTGT
**human BCL-xL**	CTCCTCTCCCGACCTGTGAT	AAGATTCTGAAGGGAGAGAAAGAGA
**human MCL-1**	GTGCAGCGCAACCACGAG	CGATTTCACATCGTCTTCGTTT
**human PI3K**	TAGCTATTCCCACGCAGGAC	TTGCTTTGAGCTGTTCTTTGTC
**human mTOR**	CCCACGTTCCTTAACGTCAT	GGCTCTTCACAAAGGACACC
**human ABL1**	TGTATGATTTTGTGGCCAGTGGAG	GCCTAAGACCCGGAGCTTTTCA
**human ß-Actin**	TCGACAACGGCTCCGGCATG	CCTCTCTTGCTCTGGGCCTCGTC

Abbreviations: BCL-2, B-cell lymphoma; BCL-xL, B-cell lymphoma – extra- large; MCL-1, myeloid leukemia cell differentiation protein-1, PI3K, phosphoinositide 3 kinase; mTOR, mechanistic target of rapamycin (formerly: mammalian target of rapamycin).
