# Supplementary material for: The pan-BCL-2-blocker obatoclax (GX15-070) and the PI3-kinase/mTOR-inhibitor BEZ235 produce cooperative growth-inhibitory effects in ALL cells
Source: Oncotarget. 2017 Jun 28;8(40):67709–22. doi: 10.18632/oncotarget.18810 (PMC5620205; doi:10.18632/oncotarget.18810)
Supplement: Supplementary file 2 [file oncotarget-08-67709-s002.doc]

**Supplementary Table 6**

**Patients´ characteristics**

| **Pat. No** | **Gender** | **Age** | **Diagnosis** | **WBC** | **Hb** | **Plt** | **Blasts** | **Blasts** | **LDH** | **Karyotype** | **BCR/ABL1** |  |
| --- | --- | --- | --- | --- | --- | --- | --- | --- | --- | --- | --- | --- |
| **No#** | **(f/m)** | **(yrs)** |  | **(G/l)** | **(g/dl)** | **(G/l)** | **(%) PB** | **(%) BM** | **(U/l)** |  | **(variant)** | **Mutation** |
| 1 | m | 25 | c-ALL (Relapse) | 29.86 | 13.9 | 197 | 41 | 87 | 826 | 46,XY,complex | - | n.t. |
| 2 | m | 37 | Mixed phenotype AL (B-Type) | 8.44 | 6.8 | 5 | 48 | 86 | 838 | 45,XY,-20/46,XY,del(20q) | - | n.t. |
| 3 | f | 24 | c-ALL | 3.98 | 8.3 | 171 | 17 | 40 | 244 | 46,XX | - | n.t. |
| 4 | f | 34 | c-ALL | 15.01 | 8.6 | 205 | 30 | 50 | 568 | 45,XX,-7,t(9:22)(q34;q11) | +(p210) | - |
| 5 | m | 20 | Mixed phenotype AL (T-Type) | 31.73 | 11.3 | 75 | 88 | 95 | 297 | 46,XY,t(2;14)(q21;q31)/46,XY) | - | n.t. |
| 6 | m | 77 | c-ALL (Relapse) | 43.91 | 9.8 | 18 | 95 | n.a. | 468 | t(9;22)(q34;q11) | +(p190) | T315I |
|  | m | 78 | c-ALL (Relapse) | 29.60 | 13 | 19 | 51 | n.a. | 591 | t(9;22)(q34;q11) | +(p190) | G255V, T315I |
| 7 | f | 32 | pre-B-ALL | 122.40 | 9.1 | 68 | 84 | 83 | 825 | 46,XX,del(9)(p13)/46,XX | - | n.t. |
| 8 | f | 70 | "Mature" T-ALL | 128.00 | 9.3 | 30 | 67 | 90 | 3168 | 46,XX,complex | - | n.t. |
| 9 | f | 31 | pre-B-ALL (Relapse) | 17.65 | 9.6 | 24 | 76 | 79 | 1830 | t(14;14) | - | n.t. |
| 10 | f | 25 | c-ALL | 16.74 | 9.4 | 12 | 69 | >95 | 235 | 46,XX,t(9;22)(q34;q11) | +(p190) | - |
| 11 | m | 40 | T-ALL (Relapse) | 40.93 | 11.6 | 125 | 64 | 75 | 1533 | 46,XY | - | n.t. |
| 12 | f | 50 | c-ALL | 37.50 | 7.6 | 135 | n.a. | >90 | 473 | 46,XX,del(9)(p21),t(9;22)(q34;q11) | +(p190) | n.t. |
| 13 | f | 67 | c-ALL | 193.43 | 7.7 | 33 | 68 | 70 | 998 | 46,XX,t(6;11)(q27;q23) | +(p210) | - |
|  | f | 68 | c-ALL (1.Relapse) | 5.19 | 10.2 | 208 | n.a. | 18-19 | 314 | 47,XX,t(6;7),+8,t(9;22) | +(p210) | T315I |
|  | f | 68 | c-ALL (2.Relapse) | 82.80 | 11.2 | 46 | 65 | n.a. | 980 | 46,XX,t(9;22),complex | +(p210) | G255L, T315I |
|  | f | 68 | c-ALL (2.Relapse) | 35.19 | 10.1 | 12 | n.a. | n.a. | 470 | n.a. | +(p210) | G255L, T315I |
| 14 | f | 18 | c-ALL | 2.82 | 11 | 133 | n.a. | 70 | 342 | 46,XX | - | n.t. |
| 15 | m | 72 | c-ALL | 96.89 | 10 | 73 | 46 | 57 | 1019 | 46,XY,t(9;22)(q34;q11.2) | +(p210) | - |
| 16 | m | 24 | pro-B-ALL | 144.10 | 10.8 | 18 | 60 | >95 | n.a. | 46,XY | - | n.t. |
| 17 | m | 38 | pre-B-ALL | 137.35 | 7.3 | 29 | 50 | n.a. | n.a. | 46,XY,t(9;22)(q34;q11.2) | +(p210) | n.t. |
| 18 | m | 22 | c-ALL | 153.8 | 6.3 | 24 | 81 | 90 | 1515 | n.a. | - | n.t. |
|  | m | 23 | c-ALL (Relapse) | 1.12 | 9.0 | 7 | n.a. | 80 | 142 | 50,XXY,complex | - | n.t. |
| 19 | m | 60 | pre-B-ALL (Relapse) | 13.16 | 11.9 | 93 | 7 | 40 | 429 | 46,XY | - | n.t. |
| 20 | f | 35 | pre-B-ALL | 155.26 | 8.6 | 52 | 67 | 90 | 555 | 46,XX,t(11;19),+9 | - | n.t. |
| 21 | f | 67 | c-ALL (Relapse) | 1.82 | 7.7 | 7 | n.a. | 75 | 412 | 46,XY | - | n.t. |
| 22 | m | 27 | c-ALL | 6.38 | 15 | 14 | 18 | n.a. | 620 | 46,XY,t(9;22)(q34;q11),complex | +(p210) | - |
| 23 | m | 72 | c-ALL | 4.12 | 7.7 | 17 | 82 | n.a. | 1337 | n.a. | + | T315I |

Pat.No, patient number; f, female; m, male; WBC, white blood count; Hb, hemoglobin; PLT, platelet count; PB, peripheral blood; BM, bone marrow; n.a., not available; n.t. not tested; -, negative for BCR-ABL1 or no mutation found
